# Clinical effectiveness of rapid tests for methicillin resistant *Staphylococcus aureus *(MRSA) in hospitalized patients: a systematic review

**DOI:** 10.1186/1471-2334-11-336

**Published:** 2011-12-12

**Authors:** Julie Polisena, Stella Chen, Karen Cimon, Sarah McGill, Kevin Forward, Michael Gardam

**Affiliations:** 1 Canadian Agency for Drugs and Technologies in Health, Ottawa, Ontario, Canada; 2 Division of Microbiology, Department of Pathology and Laboratory Science, Dalhousie University, Halifax, Nova Scotia, Canada; 3 Infection Prevention and Control, University Health Network, Toronto, Ontario, Canada

## Abstract

**Background:**

Methicillin resistant *Staphylococcus aureus *(MRSA) are often resistant to multiple classes of antibiotics. The research objectives of this systematic review were to evaluate the clinical effectiveness of polymerase chain reaction (PCR) versus chromogenic agar for MRSA screening, and PCR versus no screening for several clinical outcomes, including MRSA colonization and infection rates.

**Methods:**

An electronic literature search was conducted on studies evaluating polymerase chain reaction techniques and methicillin (also spelled meticillin) resistant *Staphylococcus aureus *that were published from 1993 onwards using Medline, Medline In-Process & Other Non-Indexed Citations, BIOSIS Previews, and EMBASE. Due to the presence of heterogeneity in the selected studies, the clinical findings of individual studies were described.

**Results:**

Nine studies that compared screening for MRSA using PCR versus screening using chromogenic agar in a hospital setting, and two studies that compared screening using PCR with no or targeted screening were identified. Some studies found lower MRSA colonization and acquisition, infection, and transmission rates in screening with PCR versus screening with chromogenic agar, and the turnaround time for screening test results was lower for PCR. One study reported a lower number of unnecessary isolation days with screening using PCR versus screening with chromogenic agar, but the proportion of patients isolated was similar between both groups. The turnaround time for test results and number of isolation days were lower for PCR versus chromogenic agar for MRSA screening.

**Conclusions:**

The use of PCR for MRSA screening demonstrated a lower turnaround time and number of isolation days compared with chromogenic agar. Given the mixed quality and number of studies (11 studies), gaps remain in the published literature and the evidence remains insufficient. In addition to screening, factors such as the number of contacts between healthcare workers and patients, number of patients attended by one healthcare worker per day, probability of colonization among healthcare workers, and MRSA status of hospital shared equipment and hospital environment must be considered to control the transmission of MRSA in a hospital setting.

## Background

Methicillin resistant *Staphylococcus aureus *(MRSA) are often resistant to multiple classes of antibiotics. In hospitals, transmission occurs from a colonized or an infected individual to others, mainly via the hands of transiently-colonized healthcare workers [1]. MRSA has been associated with many infection sites including bones and joints, lungs, and the urinary tract [2]. Bacteremia is common, possibly leading to endocarditis and osteomyelitis [2]. Hospital-acquired MRSA is typically resistant to classes of antimicrobials other than β-lactams [3].

A national MRSA prevalence study by the Association for Professionals in Infection Control and Epidemiology, Inc., conducted in 2006, determined that 46 out of every 1,000 patients in the United States were either MRSA colonized or infected [4]. The incidence of MRSA in 47 sentinel, geographically-dispersed Canadian hospitals participating in the Canadian Nosocomial Infection Surveillance Program in 2007 was 8.62 cases (2.57 infection and 5.87 colonization) per 1,000 patient admissions and 11.63 cases (3.47 infection and 7.92 colonization) per 10,000 patient-days [5]. In Europe, the 2007 incidence of MRSA bloodstream infection per 100,000 patient days ranged from 0.2 in Sweden to 2.4 in Portugal [6].

Active surveillance of MRSA is part of an infection control and preventive measure that also includes isolation, cohorting and decolonization. The effectiveness of these practices to reduce the risk of MRSA transmission in a hospital setting remains controversial [7,8].

The polymerase chain reaction (PCR) is a molecular technique in which enzymatic replication is used to amplify a short sequence of DNA. Because it is used to reproduce selected sections of DNA, the presence of MRSA is more rapidly and easily detected (up to five hours) compared with culture-based methods, which can take one to two days [9]. PCR is used to identify the SCC mec cassette that contains the mecA gene and orfX, an opening frame distinctive to *Staphylococcous aureus*[10,11]. In the US and Canada, there are three automated commercially available PCR systems that are approved for detection of MRSA: BD GeneOhm MRSA Assay, Xpert MRSA Assay, and Roche LightCycler MRSA Advanced. They are qualitative, in vitro diagnostic tests designed for direct detection of MRSA nasal colonization [12-15].

The primary research objective of this systematic review was to evaluate the clinical effectiveness of screening with PCR versus screening with chromogenic agar or no screening with regards to MRSA colonization, acquisition, transmission and infection rates, the turnaround time to report the test results and number of isolation days and inappropriate open days (patients for whom isolation precautions were not implemented and who were MRSA positive on admission) in a hospital setting.

## Methods

### Literature search strategy

Peer reviewed literature searches were conducted for the clinical review. The following bibliographic databases were searched through the OvidSP interface: Medline, Medline In-Process & Other Non-Indexed Citations, EMBASE, Biosis Previews, and CINAHL. Parallel searches were run in PubMed and the Cochrane Library. The search strategy comprised both controlled vocabulary, such as the National Library of Medicine's MeSH (Medical Subject Headings), and keywords. The main search concepts were methicillin (also spelled meticillin) resistant *Staphylococcus aureus*, and polymerase chain reaction techniques. No methodological filters were applied. Searches were restricted to articles published from January 1993 until May 2011 to focus on current PCR techniques.

### Selection criteria

Studies were eligible if they compared MRSA screening using one of the commercially available PCR tests, BD GeneOhm MRSA, Xpert MRSA and RocheLightCycler MRSA Advanced, versus screening with chromogenic agar or no screening in adult patients. The types of outcomes measured were MRSA colonization and acquisition rates, bacteremia and other infection rates (for example, wound, surgical site) caused by MRSA, MRSA transmission rate; turnaround time from admission to results telephoned, and number of isolation and inappropriate open days. Studies with no comparator or studies that were not conducted in a hospital setting were excluded.

### Selection and data extraction

Two reviewers (JP, SC) scanned the titles and abstracts that were identified during the literature search and applied the selection criteria. Data from each included trial were extracted by two reviewers (JP, SC) working independently with a structured form, and were verified for discrepancies and tabulated. Any differences were discussed and resolved by consensus. When an agreement could not be achieved, a third reviewer cast a deciding vote.

### Quality assessment

A quality assessment of randomized and non-randomized studies that measured the clinical effectiveness of MRSA screening was assessed independently by two reviewers (JP, SC), using a modified checklist by Downs and Black [16]. One item, source of funding for the study, was added to the checklist. A critical appraisal of reporting, external and internal validity, and power for each included selected study was conducted. Any differences were discussed and resolved by consensus. When an agreement could not be achieved, a third reviewer cast a deciding vote.

### Data analysis methods

Because there was great variation in the control arms, study design, and reporting of clinical findings for each study, a formal meta-analysis was not done. Instead, the studies were described individually.

## Results

### Quantity of research available

**Figure 1:**
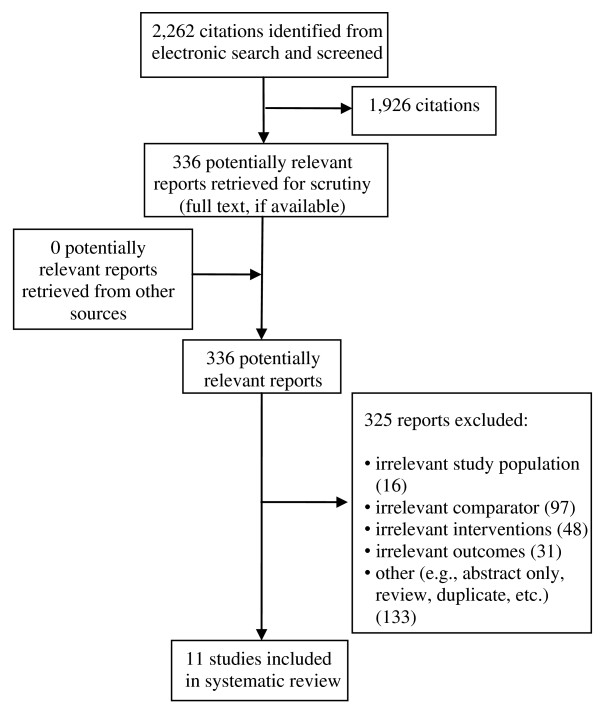
**Flowchart of Included Studies for Clinical Review**.

The literature search identified 2,262 citations. From these, 336 potentially relevant full-text articles were retrieved for further scrutiny. Eleven studies were selected for inclusion. Studies were excluded if the study participants were not limited to hospitalized patients, used an inappropriate intervention or comparators, or no comparators, were not performed in a hospital setting, did not examine hospital-acquired MRSA or measured the wrong outcomes. The PRISMA flowchart in Figure 1 details the process of the study selection.

### Study characteristics

The study characteristics appear in Table 1.

**Table 1:** Study Characteristics

First author; country; year	Number of centres; sponsor	Study design; swab; anatomical site of specimen	Number of patients and patient characteristics;	Study duration	Description of comparison arms
Hombach; Switzerland; 2010[24]	1 hospital; NR	Prospective cohort study; Copan^® ^swab; nose and groin	425 patients who i) arrived from or travelled to countries with known high rates of prevalence; ii) were transferred from LTC facilities; iii) were transferred from another health care facility; iv) were hospitalized within the previous 6 months; or v) had a history of MRSA colonization or infection	August 2007 to August 2008	Control arm: Broth-enriched (1 ml; tryptic soy broth [Becton Dickinson, Franklin Lakes, NJ, USA] supplemented with 7.5% NaCl and incubated for 24 h in ambient air 35°C. Swabs were subcultured on chromogenic agar medium (ChromIDMRSa agar; bioMérieux, Marcy l'Etoile, France) in ambient air 35°C Intervention arm 1: BD GeneOhm MRSA assay (BD, san Diego, CA, USA) performed with a SmartCycler II (Cepheid, Sunnyvale, CA, USA) according to manufacturer's instructions Intervention arm 2: Xpert MRSA assay (Cepheid, Inc., Sunnyvale, CA, USA)

Wassenberg; Netherlands; 2010[23]	14 hospitals; government and industry	Prospective cohort study; swab not specified; anterior nares, throat and perineum	1,764 patients admitted to internal medicine, paediatrics, cardiology, neurology, and other (not specified) wards were included in the study. Patients admitted to the ICU were excluded from the BD GeneOhm MRSA study.	December 2005 to June 2008	Control arm: Conventional culture (not specified) Intervention arm 1: BD GeneOhm MRSA PCR (BD Diagnostics, San Diego, CA, USA) run on the Cepheid Smart Cycler. Intervention arm 2: Xpert MRSA assay (Cepheid, Sunnyvale, CA, USA) Intervention arm 3: Chromogenic agar (MRSA-ID, bioMérieux, Marcy-l'Étoile, France)

Laurent; Belgium; 2010[17]	32-bed geriatric ward of a 390-bed tertiary-care hospital; industry	Prospective cohort study; Copan^® ^swab; nares	246 patients admitted to the geriatric ward, who presented at least one risk factor for MRSA colonization (i.e., antimicrobial therapy within the last 3 months, transfer from another hospital or from a nursing home, hospitalization in the previous year, presence of chronic wounds, past history of MRSA carriage or infection)	November 2007 to July 2008	Control arm: Chromogenic agar (CAM) MRSA Select (Bio-Rad, Marne La Coquette, France) Intervention arm: Xpert MRSA assay on a GeneExpert DX system, version 1.2 (Cepheid) according to manufacturer's instructions

Creamer; Ireland; 2010[18]	1 acute care adult tertiary care referral hospital of 700 beds; government and industry	Prospective cohort study; Copan^® ^swab; nares and groin	567 patients who were previously MRSA-positive, transferred either internally or externally, had been hospitalized during the last 18 months and/or admitted to an ICU in the past 3 months. Patients with chronic wounds, underlying skin conditions, urinary catheters, stomas, or intravascular devices other than peripheral intravenous catheters were also included.	September 2008 to February 2009 during 3 time periods: Period 1: 5-week observational period where at-risk patients for MRSA colonization were screened only with culture Period 2: 10-week period where screening specimens were processed by culture and by a rapid PCR assay Period 3: 5--week observational period where only culture was used for MRSA screening During the study period, patients in the emergency department, medical wards and surgical wards were screened.	Periods 1 and 3: Patients were screened with direct culture on MRSA*Select *chromogenic agar plates (Bio-Rad Life Science Group) Period 2: Xpert MRSA real-time PCR assay on the GeneXpert platform (Cepheid)

Snyder; US; 2010[19]	1 tertiary teaching hospital of 350 beds; none	Prospective cohort study; BBL CultureSwab; nares	Patients in ICU units, such as medical, surgical, neurological and cardiac Number of patients = NR	Study duration = NR	Control arm: BBL CHROMagar MRSA medium (C-MRSA) and swab was then inoculated into enrichment broth, BBL tryptic soy broth with 6.5% NaCl (BD Diagnostics, Sparks, MD, USA) Intervention arm: BD GeneOhm MRSA real-time PCR system (BD Diagnostics-GeneOhm, San Diego, CA, USA)

Pofahl; US; 2009[20]	1 tertiary care hospital of 761 beds; none	Prospective cohort study; swab not specified; nares	5,094 patients undergoing surgical infection prevention project procedures Targeted screening = 56,835 operations Universal screening = 35,778 operations	Period 1 (targeted screening) = January 1, 2004 Period 2 (universal screening) = February 15, 2007 Note: The end date of each study period was not reported.	Period 1: High-risk patients for MRSA carriage were screened on admission and placed in contact precaution prior to test results notification. MRSA screening test method for this phase was not specified. Period 2: All admissions were screened for MRSA using BD GeneOhm, and patients were placed in contact precaution prior to notification of test results. Positive test results were confirmed with culture-based methods.

Hardy; UK; 2009[25]	1 teaching hospital of 1,200 beds; industry	Prospective, cluster two-period cross-over trial; swab not specified; nares	10,934 patients admitted to one of the study wards [general surgery (2), thoracic (1), ear, nose and throat (1), trauma and orthopedic (2) and urology (1)] for > 24 hours = 13,952 patient ward episodes (i.e., each separate ward admission for the same patient was counted. 1,270 (9.1%) PWE were excluded from the study analysis. [32 (0.2%) had no sample taken at admission and 1,238 (8.8%) had no sample taken at all]	January 2005 to April 2007 (2-month pilot period, two 8-month crossover periods and 1-month follow-up of study patients)	Control arm: chromogenic agar (MRSA ID; Biomerieux, Marcy, l'Étoile, France) Intervention arm: BD GeneOhm MRSA (BD Diagnostics-GeneOhm, San Diego, CA, USA) Where discrepant results occurred between chromogenic agar and PCR, samples were placed into broth enrichment and then sub-cultured onto chromogenic media.

Aldeyab, UK, 2009[26]	1 hospital; none	Cluster crossover trial; Copan swabs^®^; nares, axillae and groin	Patients in medical⁄cardiology (2) and surgical (2) wards were studied. Number of patients = NR	Phase 1: October 2006 to January 2007 Phase 2: February 2007 to May 2007 2-week washout period occurred between phases	Phase 1: Patients in surgical ward were screened using IDI-MRSA assay (BD GeneOhm, Oxford, UK) and patients in the medical⁄oncology ward were screened using chromogenic agar (MRSA-ID)* Phase 2: Both wards switched screening methods*

Robicsek; US; 2008[21]	3-hospital organization; honoraria from industry	Prospective cohort study; swab not specified; nares	No active surveillance (i.e., no screening) = 39,521 ICU surveillance = 40,392 Universal surveillance = 73,427	August 2003 to April 2007	Period 1: Routine surveillance of MRSA did not occur Period 2: Nasal surveillance for MRSA colonization for ICU admissions using an in-house screening method** Period 3: Nasal surveillance for MRSA colonization for all hospital admissions on day one using BD GeneOhm

Jeyaratnam; UK; 2008[27]	1 hospital (2 sites); government	Cluster randomized crossover trial; swab not specified; nares, axillae and groin	9,608 patients were admitted to study wards: [surgical (6), elderly care (2), and oncology (2)]. (6,888 patients had full data and were eligible)	January 2006 to March 2007 (3-month baseline period, 5-month intervention period, 1-month washout period, and 5-month intervention period)	Control arm: swabs were taken on admission for culture only Intervention arm: one swab used BD GeneOhm MRSA Assay and another swab used culture Culture method: swabs were cultured in a selective broth and, after May 2006, were combined with Chromagar (Oxoid, Basingstoke, UK)

Jog; UK; 2008[22]	1 hospital; none	Prospective, cohort study; swab not specified; nares	1,462 patients admitted for cardiac surgery	October 2004 to September 2006	Control arm: chromID MRSA agar (bioMérieux, Marcy l'Étoile, France) and enrichment culture was performed after the assay procedure. For the broth enrichment, the swabs were incubated overnight in tryptic soy broth containing 6.5% NaCl at 35°C before subculture onto chromID MRSA and 5% sheep blood agar (Oxoid, Basingstoke, UK) with a 1 μg oxacillin disc. Intervention arm: Gene Ohm MRSA Test (Becton Dickinson, New Jersey, USA) run on the Cepheid Smart Cycler.

ICU = Intensive care unit; LTC = Long-term care; NR = Not reported

*All swabs were also inoculated into Robertson's cooked meat broth, incubated at 35°C for 18-24 h and were then subcultured onto MRSA-ID.

**Only the universal and no active surveillance arms were examined based on the selection criteria for the clinical review.

### Study Design

A prospective cohort design was used in eight studies,[17-24] and three studies were cluster crossover trials [25-27].

### Patient Population

Studies were conducted in surgical,[18-20,22,25,26] elderly care,[17,27] oncology,[27] and medical-cardiology,[23,26] wards in a hospital setting. In addition to the cardiology ward, Wassenberg et al. included patients admitted to intensive care units and to internal medicine, surgical, pediatrics, and neurology wards [23]. Hombach et al. did not specify the hospital wards their study [23]. The sample size ranged from 246[17] to 153,511 patients [21].

### Collection and Anatomical Site of Specimen

Four studies used Copan swabs for specimen collection and transport [17,18,24,26]. One study used the BBL CultureSwab [19]. The remaining studies did not specify the type of specimen transport system used. In all studies that reported the details of the specimen transport system, liquid Stuart medium was used. Six studies examined nasal specimens only [23]. In addition to nares, specimens were collected from the throat, axillae, perineum or groin in five other trials [18,23,24,26,27].

### Intervention and comparators

BD GeneOhmMRSA was compared with chromogenic agar in seven studies,[19-22,25-27] and Xpert MRSA was assessed in two other studies [17,18]. The clinical effectiveness of BD GeneOhmMRSA and Xpert MRSA was assessed in two studies [23,24]. Studies that evaluated LightCycler MRSA Advanced did not meet the selection criteria. Five studies compared the clinical effectiveness of PCR with chromogenic agar (MRSA ID, Biomerieux, Marcy l'Étoile, France) [23-26]. Chromogenic agar MRSA *Select *(Bio-Rad, Marne La Coquette, France) was used in two studies;[17,18] Jeyaratnam et al. included MRSA Chromoagar, Oxoid, Basingstole, UK)[27] as the comparator, and Synder et al. used BBL CHROMagar MRSA medium (C-MRSA)[19]. Two studies did not identify the test method used during specific phases [21,22]. As part of the preparation process, the specimens were broth-enriched in four studies [19,22,24,27].

### Quality assessment

The methodological quality of the studies in this systematic review was mixed. The assessment indicated that the weakest area was internal validity related to confounding. In particular, it was unclear whether patients were randomized to the intervention or control arms,[26] and if potential confounders were investigated,[21,26] and there was a lack of reporting about patients who were lost to follow-up [21,26]. In Pofahl et al.'s and Robicsek et al.'s studies, the intervention and control groups were studied during different phases over a period of approximately three years to seven years [20,21]. This study design may lead to potential biases because several factors that were related to investigator knowledge (the investigator is not blinded from the outcome variable), timing (interventions at different study phases over a long time period may lead to changes in behaviour) and proficiency (different resources or procedures may be used at different times) may affect the estimated comparisons of the clinical outcomes [21]. One study explicitly met the criteria for internal validity [27]. Patients were randomized, and there were adjustments for confounding, such as standard errors for correlation within wards. A restricted primary outcome analysis was conducted where all patients with any MRSA positive culture specimen within three months before admission, and those with MRSA positive discharge screens taken within 48 hours of negative admission swab were excluded. Patients who were MRSA positive on admission and those lost to follow-up were included in resource analysis [27].

### Data analyses and synthesis

The clinical findings that were associated with MRSA colonization, infection and transmission rates, as well as turnaround times and isolation days for screening using PCR compared with screening using chromogenic agar are reported in Table 2.

**Table 2:** Main Study Findings

First author	Outcome Measurement	Main study findings		
**MRSA colonization**				

Hardy[25]		**PCR**		**Chromogenic agar**
	
	PWE of MRSA positive on admission	266 (4.4%)		187 (2.8%)
	
	PWE of acquired MRSA	111 (1.9%)		157 (2.4%)
	
	Rate ratio of MRSA acquisition rate	1.49; 95% CI: 1.115-2.0003		

Aldeyab[26]		**PCR**		**Chromogenic agar**
	
	1,000 bed-days in surgical ward	20.0		22.1
	
	1,000 bed-days in medical/cardiology ward	11.8		20.3
	
	Average monthly cases of MRSA⁄100 patient-admissions in surgical ward	6.8 (range: 3.6 to 15)		7.3 (range: 5.4 to 9.3)
	
	Average monthly cases of MRSA⁄100 patient-admissions in medical/cardiology ward	9.0 (range: 3.3 to 12.2)		7.3 (range: 6.1 to 9.8)

Jeyaratnam[27]		**PCR**		**Chromogenic agar**
	
	Proportion of patients who acquired MRSA	2.8% (n = 99) (95% CI: 2.26 to 3.34)		3.2% (n = 108) (95% CI: 2.6 to 3.79)
	
	Acquisition rate⁄1,000 patient-days	4.4		4.9
	
	Incidence rate ratio per 1,000 patients days at risk	0.90; 95% CI: 0.69 to 1.2		
	
	Unadjusted odds ratio	0.88; 95% CI: 0.52 to 1.46		
	
	Adjusted odds ratio	0.91; 95% CI: 0.61 to 1.34		

**MRSA infection**				

Pofahl[20]		**Universal screening with PCR**		**Targeted screening**
	
	Infection rate per 100 procedures	0.09		0.23
	
	Infection rate per 100 procedures among patients undergoing orthopedic surgery	0.00		0.03
	
	Proportion of MRSA SSI	7.1%		11.6%

Robiscek[21]		**Universal surveillance with PCR**		**Baseline (i.e., no surveillance)**
	
	MRSA infections⁄10,000 patient-days	-5.0 (95% CI: -6.6 to -3.5)		8.9 (95% CI: 7.6 to 10.4)
	
	Change in MRSA infections from baseline to universal surveillance	-69.6% (95% CI: -89.2% to -19.6%)		
	
	Change in MRSA bacteremia from baseline to universal surveillance per 10,000 patient-days	-1.1 (95% CI: -1.9 to -0.2)		

Jeyaratnam[27]		**PCR**		**Chromogenic agar**
	
	Number of patients with wound infections	21		22
	
	Odds ratio of wound infections	0.91; 95% CI: 0.48 to 1.7		
	
	Number of MRSA bacteremia cases	1		2
	
	Odds ratio of MRSA bacteremia	0.49; 95% CI: 0.01 to 9.1		

Jog[22]		**12-months post MRSA screening**		**12-months prior to MRSA screening**
	
	SSI rate	2.22%		3.30%

**MRSA transmission rate**				

Jeyaratnam[27]		**PCR**		**Chromogenic agar**
	
	MRSA transmission rate	0.33		0.36
	
	Incidence rate ratio	0.85; 95% CI: 0.64 to 1.12		

**Turnaround time from admission to results reported**				

		**PCR**		**Chromogenic agar**

Hombach[23]		**BD Gene Ohm MRSA**	**Xpert MRSA**	
	
	Median transport time (from collection to arrival at the laboratory)	4 hours and 25 minutes	4 hours and 25 minutes	4 hours and 25 minutes
	
	Median collection time (includes administration and accumulation of specimens to utilize the master mix to full capacity-for BD GeneOhm only)	6 hours and 55 minutes	1 hour and 5 minutes	Directly inoculated
	
	Median analysis time (includes DNA extraction for BD GeneOhm MRSA and Gene Xpert)	5 hours and 40 minutes	2 hours and 20 minutes	54 hours and 30 minutes
	
	Median time to reporting from sampling to reporting of test results	17 hours	7 hours and 50 minutes	68 hours and 50 minutes
	
		**PCR**		**Chromogenic agar**
		
		**BD Gene Ohm MRSA**	**Xpert MRSA**	

Wassenberg[23]	Time from start of isolation to delivery of specimen to laboratory	8.2 hours (IQR: 1.3 to 17.0)	5.0 hours (IQR: 1.0 to 16.6)	4.8 hours (IQR: 0.7 to 14.6)
	
	Time from arrival in the laboratory to definite result	3.6 hours (IQR: 2.2 to 6.7)	2.0 hours (IQR: 1.5 to 3.3)	Not reported

Laurent[17]		**PCR**	**Chromogenic agar**	
	
	Median turnaround time	1.9 hours (IQR: 1.4 to 4.2 hours)	66.9 hours (IQR: 50.9 to 67.9 hours)	

Creamer[18]		**PCR**	**Chromogenic agar**	
	
	Mean overall turnaround time	13.2 hours	46.2 hours	
	
	Mean overall turnaround time for MRSA-positive specimens	17.1 hours (range: 2.0 to 75.8 hours)	53.9 hours (range: 26.0 to 123.8 hours)	

Snyder; US; 2010[19]		**PCR**	**Chromogenic agar**	
	
	Average time to report MRSA-positive test results	17.4 hours (range: 4.12 to 31.1 hours)	28.1 hours (range: 13.9 to 49.6 hours)	
	
	Average time to report MRSA-negative test results	14.4 hours (range: 3.12 to 33.8 hours)	51.3 hours (range: 34.32 to 65.95 hours)	

Hardy [25]		**PCR**	**Chromogenic agar**	
	
	Mean turnaround time	0.9 days	2.3 days	

Aldeyab[26]		**PCR**	**Chromogenic agar**	
	
	Median time interval in surgical ward	19.3 hours (IQR:13.8 to 23)	51.8 hours (IQR: 44.4 to 69)	
	
	Median time interval in medical/cardiology ward	22.7 hours (IQR:19.8 to 23.8 hours)	42.2 hours (IQR: 40.3 to 69.6 hours)	

Jeyaratnam[27]		**PCR**	**Chromogenic agar**	
	
	Median turnaround time	21.8 hours (IQR: 17.95 to 25.4)	46.4 hours (IQR: 39.1 to 66.1)	

**Isolation days**				

Wassenberg[23]		**PCR**		**Chromogenic agar**
		
		**BD Gene Ohm MRSA**	**Xpert MRSA**	
	
	Time from definite test result to discontinuation of isolation	0.2 hours (IQR: 0 to 0.5)	0.3 hours (0.2 to 0.5)	0.2 hours (0 to 0.5)
	
	Time from start of isolation to definite test result	17.8 hours (5.0 to 24.2)	14.0 hours (3.4 to 21.2)	31.9 hours (24.7 to 41.5)
	
	Duration of isolation	19.7 hours (6.0 to 34.6)	16.1 hours (4.0 to 24.7)	30.0 hours (24.2 to 43.0)

Jeyarathnam[27]		**PCR**	**Chromogenic agar**	
	
	Number of inappropriately isolated days	277	399	

**Open days**				

Jeyarathnam[27]		**PCR**		**Chromogenic agar**

	Number of inappropriately open days	351		389

IQR = Inter-quartile range; OR = Odds ratio; PWE = Patient ward episodes; SSI = Surgical site infection

### MRSA colonization and acquisition

Three studies compared the MRSA colonization and acquisition rates between screening with PCR (BD GeneOhm) and screening with chromogenic agar [25-27]. The overall results suggested that patients screened using PCR are less likely to transmit MRSA in a hospital setting compared with patients screened using chromogenic agar.

Hardy et al. measured the acquisition rate as the ratio of the number of patients acquiring MRSA in one study ward to the number of MRSA positive patients on admission [25]. Patients in the chromogenic agar arm were 1.49 times more likely to acquire MRSA compared with those in the PCR arm. The study also found the MRSA incidence rates per 100 bed-days to be lower for patients who were screened using PCR compared with patients who were screened using chromogenic agar (0.286 versus 0.410) [25].

Aldeyab et al. reported a lower MRSA incidence rate per 1,000 bed-days for screening with PCR versus screening with chromogenic agar among patients in the surgical ward (20.0 versus 22.1). The difference was more pronounced in the medical-cardiology ward (11.8 versus 20.3) [26].

Jeyaratnam et al. found fewer patients acquired MRSA (n = 99; 2.8%) (95% CI: 2.26 to 3.34) when PCR screening was used compared with screening using chromogenic agar (n = 108; 3.2%) (95% CI: 2.6 to 3.79) [27]. The MRSA unadjusted and adjusted acquisition rates were similar between both groups (unadjusted odds ratios = 0.88; adjusted odds ratio = 0.91) [27]. In both instances, the 95% confidence intervals (CIs) were wide, so the results must be interpreted with caution.

### MRSA infection

Jeyaratnam et al. found MRSA wound infections in 21 patients who were screened using PCR (BD GeneOhm) versus 22 patients who were screened using chromogenic agar [odds ratio (OR): 0.91; 95% CI: 0.48 to 1.7)] [27]. In this study, one MRSA bacteremia case was found in the PCR arm and two were found in the chromogenic agar arm (OR: 0.49; 95% CI: 0.01 to 9.1). The results must be interpreted with great caution given the wide CIs.

Two studies found lower rates of MRSA surgical site infections (SSIs) with universal screening using PCR (BD GeneOhm) versus no screening [20,22]. Pofahl et al. also reported a decrease in the proportion of MRSA SSIs from 11.6% in the no screening group compared with 7.1% in the universal screening group [20]. The reduction of MRSA SSIs was more significant in patients undergoing orthopedic surgery (p = 0.04) compared with patients undergoing cardiac surgery or a hysterectomy.

Robiscek et al. presented the absolute difference in infections per 10,000 patient-days between no screening and universal surveillance with PCR of -5.0 (95% CI: -6.6 to -3.5)] [21]. The study also found a decrease in the prevalence density of MRSA bacteremia from baseline to universal surveillance (absolute reduction: -1.1 per 10,000 patient-days; 95% CI: -1.9 to -0.2) [21].

### MRSA transmission

Jeyaratnam et al. reported a lower MRSA transmission rate among patients who were screened using PCR (0.33) versus patients who were screened using chromogenic agar (0.36) (incidence rate ratio: 0.85; 95% CI: 0.64 to 1.12) [27]. Since the 95% CI crosses the value of 1, the results are not statistically significant.

### Turnaround time from admission to results reported

The major promise of PCR is the lower turnaround time from admission to results reported. All studies reported a lower turnaround time for screening with PCR (BD GeneOhm MRSA) versus screening with chromogenic agar. The mean turnaround time ranged from 13.2 hours to 21.6 hours with PCR (BD GeneOhm) versus 46.2 hours to 79.2 hours using chromogenic agar, across all studies [19,25-27].

The median turnaround time was less for screening using Xpert MRSA versus screening using chromogenic agar for definitive test results (1.9 hours versus 66.9 hours) in one study[17] and the mean overall turnaround time for Xpert MRSA was 17.1 hours compared with 53.9 hours with chromogenic agar in another study [18]. The median and mean turnaround times were calculated differently in each study, which may partially explain the discrepancy in the results. For instance, Creamer et al. reported a mean turnaround time of 2.6 hours for screening with PCR if the time spent on specimen collection to the arrival of the specimen in the laboratory was not considered [18].

As seen in Table 2, Wassenburg et al. and Hombach et al. reported the shortest turnaround times from the specimen collection to a definite a test result with XpertMRSA versus BD GeneOhm MRSA and chromogenic agar [23,24]. In one study, the arrival of the specimens were longest for BD GeneOhm MRSA compared with the other test. The authors attributed the longer transportation of specimens to a laboratory with BD GeneOhm MRSA versus the other tests to a learning effect but did not provide further details [23,24]. Moreover, fewer hospitals in the study used Xpert MRSA and chromogenic agar, impacting the median transportation across comparator arms. The median transportation time was similar across all screening tests in another study,[24] but the analysis time was the greatest with chromogenic agar (chromogenic agar = 54 hours and 30 minutes versus BD GeneOhm MRSA = 5 hours and 40 minutes versus Xpert MRSA = 2 hours and 20 minutes) [24]. The laboratory time was longer with BD GeneOhm MRSA versus Xpert MRSA since the master mix had to be filled to the capacity with specimens prior to its use [23].

### Isolation and open days

According to the study by Jeyaratnam et al., the number of inappropriately isolated days (patients who were pre-emptively nursed with MRSA precautions but were not MRSA positive on admission) was lower in the PCR arm compared with the chromogenic agar arm (277 versus 399), but the proportion of patients who were pre-emptively isolated was similar between the comparison arms (5% versus 4.7%) [27]. The number of inappropriate open days (patients for whom isolation precautions were not implemented and were MRSA positive on admission) was 351 for screening with PCR and 389 for screening with chromogenic agar [27].

According to Wassenberg et al., a shortened duration of isolation in hours was observed with Xpert MRSA, closely followed by BD GeneOhm MRSA. The duration of isolation for chromogenic agar was also doubled compared with Xpert MRSA (30.0 hours versus 16.1 hours) [23].

## Discussion

Eleven studies on the clinical effectiveness of screening with PCR versus screening with chromogenic agar or no screening were included in our systematic review. Overall, MRSA colonization, infection, and transmission rates were lower using PCR for screening versus chromogenic agar, but the possibility of a null difference could not be excluded based on the 95% CIs in most cases. One study detected a decrease in the prevalence of MRSA infection and bacteremia between universal screening with PCR compared with no screening [21]. In addition to placing colonized patients in isolation rooms, attempts were made to decolonize them. Therefore, it is difficult to discern the relative benefit of isolation compared with decolonization using this strategy. Potential biases that are related to study design may have affected the outcome variables.

Harbarth et al [28]. conducted a prospective, interventional cohort study using a crossover design to measure the effect on MRSA infection rates in surgical patients at admission, when universal screening is used. Standardized infection control measures (for example, hand hygiene) were compared with same-day multiplex in-house PCR plus standardized infection control measures. The results showed that same-day, universal screening did not reduce the MRSA infection rate in large, surgical wards compared with standardized infection control measures (adjusted incidence rate ratio: 1.20; 95% CI: 0.85 to 1.69), suggesting that evidence in support of MRSA universal screening at admission is inconclusive [28]. The authors noted several limitations, including no randomization of individual wards to receive interventions, a passive post-discharge MRSA surveillance of MRSA surgical site infection and no confirmation of positive test results using PCR with culture-based methods. The authors also stated that most of these limitations were resolved because of their crossover design [28].

The turnaround time from admission to screening test results being telephoned,[26,27] and the number of isolation days were lower for screening using PCR compared with screening using chromogenic agar,[27] but the large discrepancies reported between the tests is surprising since most chromogenic media are intended to be read at 24 hours. Two studies observed the shortest turnaround time with Xpert MRSA versus BD GeneOhm MRSA and chromogenic agar [23,24]. Additional factors that may influence the turnaround times are the infrastructure of the laboratory (in-house or reference) that will provide the service; prevalence rate of MRSA in hospital setting and number of specimens that will be processed; available laboratory hours; and the laboratory staff on-hand to process the screening test [7,23,24]. Despite the lower turnaround time for screening with PCR, two studies did not a find a significant difference in the MRSA transmission and acquisition rates between screening with PCR and screening with chromogenic agar [26,27]. Other interventions, such as preemptive isolation, may influence the transmission and acquisition rates in a hospital setting [25]. One study compared the diagnostic performance of chromogenic agar, BD GeneOhm MRSA and LightCycler MRSA advanced for patients in hospitals, nursing homes, extended-care facilities, and dialysis units and medical staff [29]. The authors reported that the lab technologist hands-on time for an average test batch of 25 samples using LightCycler MRSA advanced was 41 minutes compared with 75 minutes for BD GeneOhm MRSA [29]. The mean processing time was 118.5 ± 17.2 minutes for LightCycler MRSA advanced versus 137.2 ± 28.7 minutes for BD GeneOhm MRSA [29].

Even though BD GeneOhm MRSA and Xpert MRSA are PCR technologies, there are differences between the two tests. First, Xpert MRSA is automated using the Cepheid GeneXpertDx System, so screening can be performed in various health care settings, such as clinical laboratory or as point-of-care testing;[13] thus, potentially reducing the turnaround time of MRSA colonization identification [30]. Second, the screening test method can be performed health workers in a clinical laboratory or near the site of patient care [30]. The use of BD GeneOhm MRSA involves batch processing and is validated to run on the Cepheid Smart Cycler System, which is typically done in a laboratory by a medical technologist [31]. The turnaround time is approximately 72 minutes or less using Xpert MRSA compared with two hours using BD GeneOhm MRSA, depending on the batch size [31]. Although four different types of chromogenic agar were used across the studies, the clinical findings compared with PCR tests were consistent for all chromogenic agar tests.

The selection of studies and the corresponding quality assessment in the current clinical review were conducted independently by two reviewers to reduce the risk of bias. Few studies were of very high quality. For instance, the risk of confounding was greater among studies measuring the clinical effectiveness of screening using PCR with no randomization,[26] among studies that did not describe potential confounders or whether they were investigated,[21,26] and among studies that failed to state if patients who were lost to follow-up were taken into account [21,26].

Our systematic review provides insufficient evidence on the clinical effectiveness of PCR for MRSA screening in hospitalized patients according to the number and quality of studies identified in the literature. The evidence is scarcer for screening with Xpert MRSA. High-risk populations were studied, and none of the selected studies included a pediatric population. One important question relates to the most cost-effective screening test for MRSA in hospitalized patients. While this review did not examine this issue in detail, the authors will assess the cost-effectiveness of PCR tests versus chromogenic agar in a hospital setting in a separate study.

Screening is only one component of a MRSA infection-control program and it is difficult to accurately determine its relative contribution to overall control. The prevention of MRSA transmission includes measures such as hand hygiene, use of disinfectants, topical antibiotics and use of isolation procedures (consisting of the use of single rooms and the use of gowns and gloves with or without masks during all patient contact). Decolonization regimens for patients with MRSA in their nares include topical mupirocin alone or in combination with orally administered drugs (for example, rifampin in combination with trimethoprim-sulfamethoxazole or ciprofloxacin) and antimicrobial soap for bathing. These treatments are usually limited to MRSA outbreaks or settings with high-prevalence [32]. Evidence is vague concerning the influence of MRSA screening with PCR compared with other infection control strategies or changes in infection control policies on the MRSA transmission rates. Although behaviors are a challenge to measure, future studies with this focus may offer more comprehension on the optimal infection control strategy for MRSA in a hospital setting.

## Conclusions

Our systematic review found small differences in the MRSA colonization, infection, and transmission rates between screening using PCR and screening using chromogenic agar, but the turnaround time and number of isolation days were lower for screening with PCR versus screening with chromogenic agar. This difference was the most pronounced with Xpert MRSA. To contain MRSA transmission in the hospitals, factors such as the number of contacts between healthcare workers and patients, number of patients attended by one healthcare worker per day, probability of colonization among healthcare workers, and MRSA status of hospital shared equipment and hospital environment must be considered.

## Competing interests

The authors declare that they have no competing interests.

## Authors' contributions

JP led the systematic review and preparation of the manuscript.

SC participated in the systematic review and contributed to the preparation of the manuscript.

KC contributed to and reviewed the draft versions of the report.

SM developed and ran the literature search and she reviewed draft versions the manuscript.

KF contributed to and reviewed the draft versions of the report

MG contributed to and reviewed the draft versions of the report

All authors read and approved the final manuscript.

## Pre-publication history

The pre-publication history for this paper can be accessed here:

http://www.biomedcentral.com/1471-2334/11/336/prepub
